# CHIP promotes Wnt signaling and regulates Arc stability by recruiting and polyubiquitinating LEF1 or Arc

**DOI:** 10.1038/s41420-020-00394-9

**Published:** 2021-01-11

**Authors:** Yuchun Liu, Yao Sun, Yonghui Huang, Kang Cheng, Yuming Xu, Qingnan Tian, Shoutao Zhang

**Affiliations:** 1 Department of Neurology, The First Affiliated Hospital of Zhengzhou University, Zhengzhou, Henan China; 2 School of LifeSciences, Zhengzhou University, Zhengzhou, Henan China; 3 Henan Neurodevelopment Engineering Research Center for Children, Children’s Hospital Affiliated to Zhengzhou University, Zhengzhou, Henan China

**Keywords:** Cell biology, Diseases

## Abstract

The carboxyl terminus of Hsc70-interacting protein (CHIP), an E3 ubiquitin ligase, participates in many cellular processes such as protein degradation, trafficking, autophagy, apoptosis, and multiple signaling transductions. The mutant of CHIP (p.T246M) causes the spinocerebellar autosomal recessive 16 (SCAR16), a neurodegenerative disease characterized by spinocerebellar atrophy. Previous studies have shown that Wnt signaling and activity-regulated cytoskeleton-associated protein (Arc) play important roles in neurodegenerative diseases. However, the mechanisms by which CHIP regulates Wnt signaling and the stability of Arc that may affect SCAR16 are still unclear. We show that overexpression of CHIP promoted the activation of Wnt signaling, and enhanced the interaction between LEF1 and β-catenin through heightening the K63-linked polyubiquitin chains attached to LEF1, while the knockdown of CHIP had the opposite effect. Moreover, we verified that Wnt signaling was inhibited in the rat models of SCAR16 induced by the CHIP (p.T246M) mutant. CHIP also accelerated the degradation of Arc and regulated the interaction between Arc and GSK3β by heightening the K48- or K63-linked polyubiquitin chains, which further potentiated the interaction between GSK3β and β-catenin. Our data identify that CHIP is an undescribed regulator of Wnt signaling and Arc stability which may be related to the occurrence of SCAR16.

## Introduction

The carboxy-terminus of HSC70-interacting protein (CHIP), encoded by the STUB1 gene, is an E3 ubiquitin ligase containing a three tetratricopeptide repeat domains, and a U-box domain displaying E3 ubiquitination ligase activity which participates in many vital processes to modulate protein degradation, cell proliferation, metastasis, and tumor progression^1^. Previously, CHIP is reported to regulate the ubiquitination and degradation of RIPK3, PTEN, HIF1A, TRAF2/6, and Smad1/Smad4 to regulate multiple signal transductions like necroptosis, autophagy, apoptosis, NF-κB pathway, and TGF-β pathway^2–9^. It also interacts with Tau, Parkin, α-Synuclein, LRRK2, Ataxin1, and Ataxin3 which involve in the pathogenesis of various neurodegenerative diseases^10–15^. Other reports suggest that the mutant of CHIP (p.T246M) causes the spinocerebellar autosomal recessive 16 (SCAR16)^16,17^. SCAR16, also known as Gordon Holmes Syndrome, is a form of autosomal recessive spinocerebellar ataxia and accompanied by hypogonadism^18,19^. To date, there is no clinically effective treatment of SCAR16. Therefore, it remains to be fully elucidated of the detailed pathogenic mechanism of SCAR16 caused by CHIP (p.T246M) mutant.

The canonical Wnt signaling pathway plays important role in embryo development, tumorigenesis, and the occurrence of neurodegenerative diseases^20,21^. In the absence of Wnt ligand, β-catenin is tightly regulated by the adenomatous polyposis (APC), AXIN, casein kinase 1 (CK1), glycogen synthase kinase 3 (GSK3β), and β-TrCP. When Wnt glycoprotein ligands binding to Frizzled transmembrane receptors and LDL receptor-related protein 6 (LRP6), β-catenin is stabilized and translocated to the nucleus where it forms a complex with lymphoid enhancer factor (LEF1), then activates the transactivation of Wnt target genes such as *Myc*, *CyclinD1*, and *Axin2*. The dysregulation of Wnt signaling can lead to the onset and development of neurodegenerative diseases, such as Alzheimer’s, Parkinson’s, and Huntington^22^. However, whether CHIP participates in the regulation of Wnt signaling and further influences the occurrence of SCAR16 remains to be resolved.

Activity-regulated cytoskeleton-associated protein (Arc) emerges as an immediate-early gene that participates in various forms of synaptic plasticity. It has a significant effect on the formation of long-term memory and maintenance of neuronal circuit homeostasis relying on the endocytosis of AMPA-type glutamate receptors^23^. According to reports, RNF216 and Ube3a can tag Arc for ubiquitination and degradation relating to cognitive dysfunction^24–27^. GSK3α and GSK3β can catalyze Arc phosphorylation and degradation which further contribute to synaptic plasticity and the plasticity of dendritic spines^28^. These previous studies suggest that the instability of Arc may be related to cognitive disorders. Meanwhile, RNF216 is one of the pathogenic genes of SCAR16^29^, leading to a hypothesis that CHIP may regulate Wnt signaling and disturbs Arc stability which may affect the occurrence of SCAR16.

Here, our study identified that CHIP is a positive regulator of Wnt signaling and assists the degradation of Arc. We found that cytoplasmic β-catenin was reduced in the rat models of SCAR16 induced by the CHIP (p.T246M) mutant. Overexpression of CHIP increased the activation of Wnt signaling, and promoted β-catenin nucleus accumulation by enhancing the interaction between LEF1 and β-catenin through heightening the K63-linked ubiquitination of LEF1. Meanwhile, CHIP also influenced Arc stability by heightening its K48-linked ubiquitination, and altered the interaction of Arc-GSK3β-β-catenin complex by increasing Arc K63-linked ubiquitination. Therefore, our study discovers a previously unrecognized role of CHIP in the regulation of Wnt signaling and Arc stability which may be one of the pathogenic mechanisms of SCAR16.

## Results

### CHIP promotes the activation of Wnt signaling and the degradation of Arc

**Fig. 1:**
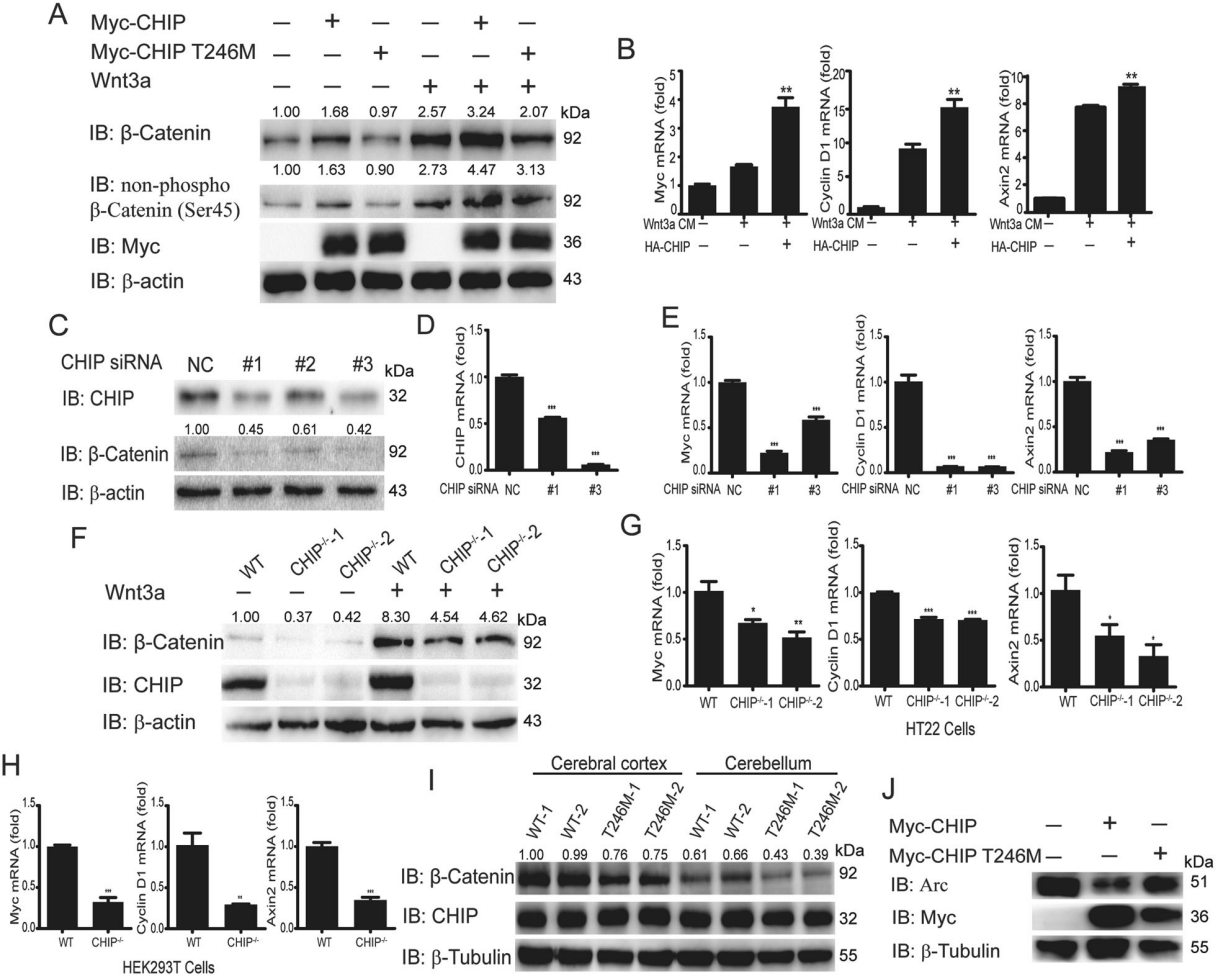
CHIP promotes the activation of Wnt signaling and the degradation of Arc. **A** CHIP increased the protein levels of β-catenin and non-phospho-β-catenin detecting by immunoblotting. HEK293T cells were transfected with an empty vector, Myc-CHIP, or Myc-CHIP T246M, and stimulated with Wnt3a (40 ng/ml) for 2 h. **B** HEK293T cells were transfected with an empty vector or HA-CHIP, and stimulated with Wnt3a CM or Ctrl-CM for 12 h. Wnt target genes (*Myc*, *Cyclin D1*, and *Axin2*) expression were analyzed by quantitative real-time PCR. **C**–**D** The knockdown efficiency of CHIP-specific siRNAs in protein level (**C**) and mRNA level (**D**). **C**, **E** Knockdown of CHIP reduced endogenous β-catenin detecting by immunoblotting (**C**), and inhibited Wnt target genes (*Myc*, *Cyclin D1*, and *Axin2*) expression detecting by quantitative real-time PCR (**E**) in SHSY5Y cells after CHIP-specific siRNAs transfection. **F** Western blot analysis of endogenous β-catenin in WT and CHIP^−/−^ HT22 cells with Wnt3a stimulation or not. **G**–**H** *Myc*, *Cyclin D1*, and *Axin2* mRNA abundance were analyzed by quantitative real-time PCR in WT and CHIP^−/−^ HT22 cells (**G**), as well as WT and CHIP^−/−^ 293T cells (**H**). **I** Immunoblot analysis of the protein level of β-catenin in cerebral cortex and cerebellum from the rat models of SCAR16 induced by CHIP T246M mutant or WT rats. **J** Immunoblotting analysis of the protein level of Arc after transfected with Myc-CHIP WT or Myc-CHIP T246M in SHSY5Y cells. All results were representative of three independent experiments. The graphs showed the means ± SD of three independent experiments. *P < 0.05, **P < 0.01, and ***P < 0.001 (Student’s *t*-test).

Recent studies have reported that CHIP (p.T246M) mutant results in the occurrence of SCAR16^16,17^. Because the Wnt signaling pathway has been reported to play important roles in the regulation of neurodegenerative diseases, we first detected the function of CHIP in the transduction of Wnt signaling. We transfected HEK293T cells with CHIP or CHIP T246M, and stimulated the cells with Wnt3a. As shown in Fig. 1A, CHIP increased the protein level of β-catenin and active-β-catenin, while CHIP T246M mutant hardly had any effect. CHIP significantly enhanced the protein level of β-catenin and active-β-catenin under the Wnt3a stimulation. Meanwhile, in real-time PCR assays, we found that CHIP increased the expression levels of Wnt target genes, such as *Myc*, *Cyclin D1*, and *Axin2* (Fig. 1B). Then to detect whether endogenous CHIP had the same effect on the regulation of Wnt signaling, we generated three pairs of siRNAs specific for CHIP, two of which validly inhibited the expression of CHIP at protein and mRNA levels (Fig. 1C, D). Further analyses showed that knockdown of CHIP by siRNA significantly decreased the protein level of β-catenin (Fig. 1C), and reduced the mRNA levels of Wnt target genes in SHSY5Y cells (Fig. 1E). At last, we obtained two CHIP^−/−^ HT22 cell lines and one CHIP^−/−^ HEK293T cell line using the CRISPR/Cas9 system and validated CHIP knockout cell lines by western blotting and sequencing (Fig. S1A–D). We found that knockout of CHIP showed a similar function as the knockdown in Wnt signaling (Fig. 1F–H). In addition, we found knockout of CHIP inhibited the migration and proliferation of HT22 cells through wound healing assay and MTT assay (Fig. S1E–G). These results revealed that CHIP had the function of maintaining cell proliferation and migration. CHIP (p.T246M) mutant lost its E3 ligase function, which might decrease the activation of Wnt signaling leading to cerebellum atrophy. We finally confirmed that Wnt signaling was inhibited in the rat model of SCAR16 induced by CHIP T246M mutant, suggesting that CHIP mutant might trigger SCAR16 by attenuating the activation of Wnt signaling (Fig. 1I). What’s more, CHIP was more significantly enriched in neural cell lines and brain tissues, correlating with the occurrence of neurodegenerative diseases (Fig. S1H, I). Hence, these data implied that CHIP was involved in the regulation of Wnt signaling.

It has been reported that the pathogenesis of SCAR16 characterized by cognitive decline and dementia is also associated with Arc that is critical for synaptic plasticity and memory^23^. For example, RNF216, which is another pathogenic gene of SCAR16, could disrupt the ubiquitination and degradation of synaptic protein Arc^25^. As GSK3α and GSK3β participate in the phosphorylation and degradation of Arc correlating Arc with Wnt signaling^28^, therefore, we want to detect whether CHIP regulates the stability of Arc. We found that CHIP facilitated the degradation of Arc, which was in consistent with the function of RNF216, while CHIP T246M had a slight function (Fig. 1J). These results suggested that CHIP promoted the activation of Wnt signaling and degradation of Arc.

### CHIP accelerates the nucleus accumulation of β-catenin

**Fig. 2:**
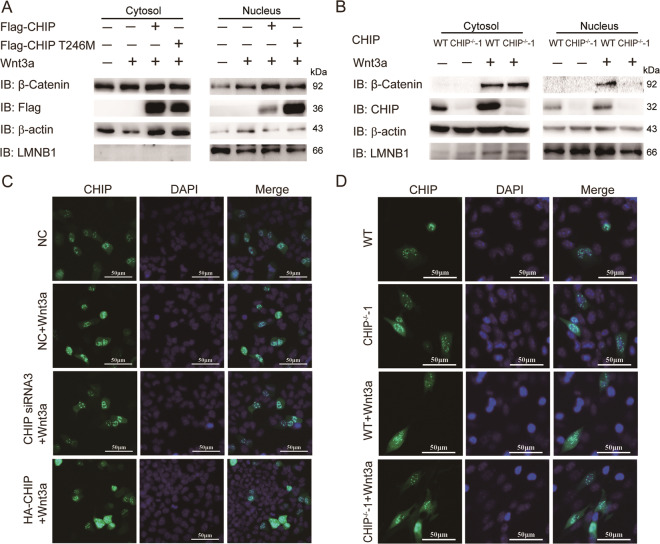
CHIP accelerates the nucleus accumulation of β-catenin. **A** HEK293T cells were transfected with Flag-CHIP or Flag-CHIP T246M, and stimulated with Wnt3a (40 ng/ml) for 4 h. Western blot analyzed the endogenous β-catenin from cytosolic and nuclear fractions. **B** Western blot analysis of endogenous β-catenin from the cytoplasm and nuclear fractions of WT or CHIP^−/−^ HT22 cells. **C**–**D** Fluorescence analysis was performed to detect the distribution of β-catenin. HEK293T cells were transfected with control siRNA (NC), CHIP-specific siRNA 3 (40 nM), or HA-CHIP along with β-catenin-GFP, and then stimulated with Wnt3a (40 ng/ml) for 4 h (**C**). WT and CHIP^−/−^ HT22 cells were transfected with β-catenin-GFP, and stimulated with Wnt3a (**D**). Fluorescence analysis of β-catenin nuclear localization with GFP (green), DNA was visualized with DAPI (blue). All results were representative of three independent experiments.

The nucleus translocation of β-catenin is an important characteristic in the activated Wnt signaling pathway. To investigate the role of CHIP in Wnt pathway, we detected the protein level of β-catenin in nuclear. We transfected HEK293T cells with plasmids of CHIP WT or CHIP T246M, stimulated cells with Wnt3a, and then detected the abundance of β-catenin in cytosolic and nuclear fractions by immunoblotting. CHIP accelerated the nucleus translocation of β-catenin, while CHIP T246M had a reduced function (Fig. 2A). In the Wnt3a-treated CHIP^−/−^−1 HT22 cell, we found the protein level of β-catenin was reduced in the nuclear fraction (Fig. 2B). To further determine whether CHIP accelerates the nucleus accumulation of β-catenin, we processed CHIP and detected β-catenin in the nucleus by fluorescence analysis. The results showed that overexpression of CHIP increased the nucleus β-catenin, nevertheless the knockdown or knockout of CHIP decreased the amount of β-catenin in the nucleus (Fig. 2C, D). Thus, these data indicated that CHIP enhanced the nucleus accumulation of β-catenin.

### CHIP interacts with LEF1 and Arc

**Fig. 3:**
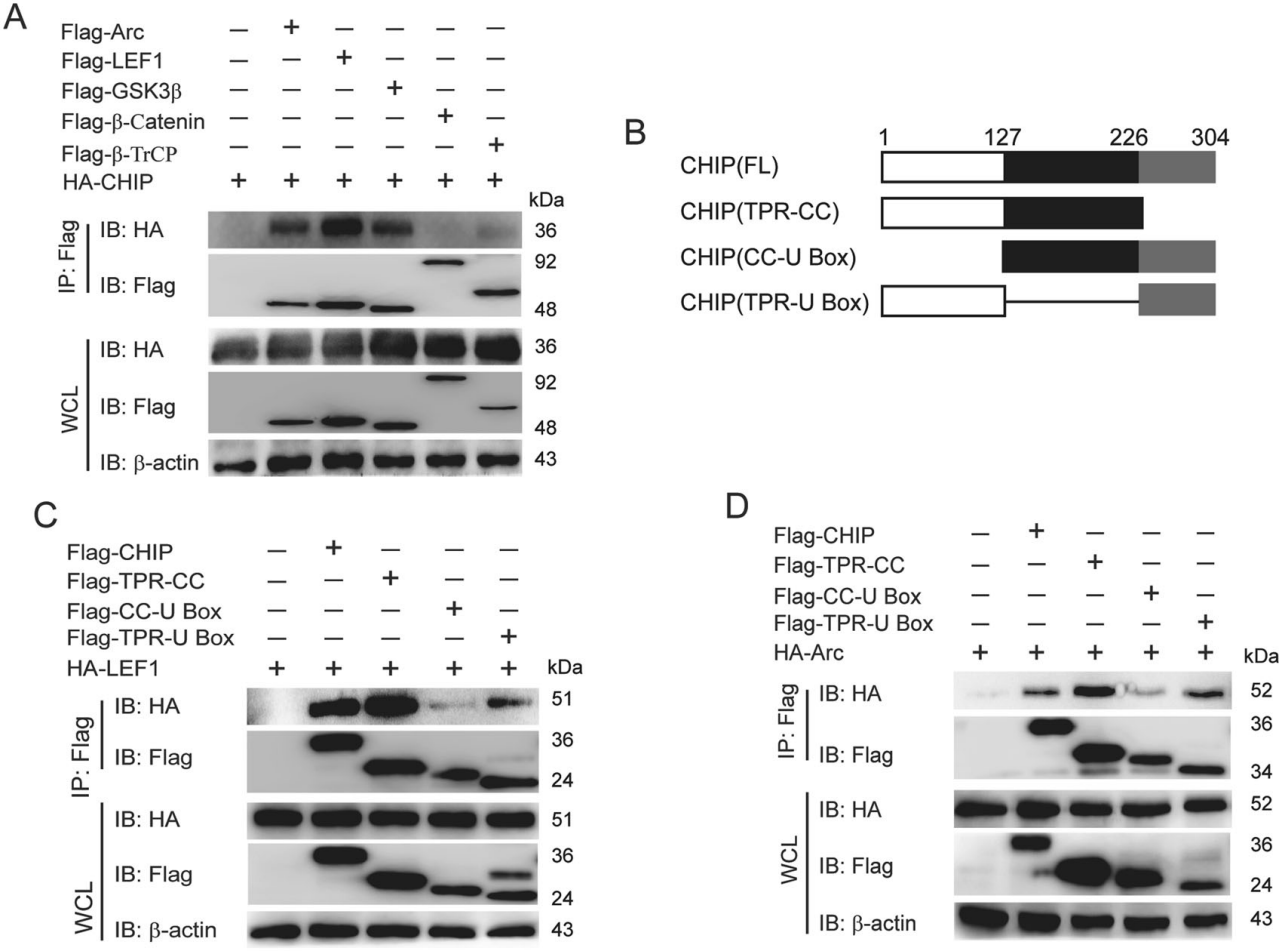
CHIP interacts with LEF1 and Arc. **A** HEK293T cells were transfected with HA-CHIP and Flag-Arc, Flag-LEF1, Flag-GSK3β, Flag-β-catenin, or Flag-β-TrCP, and the cell lysates were detected by co-immunoprecipitation and immunoblotting. **B** The domain structure of CHIP. Numbers indicated the amino acid position in construct. **C**–**D** HEK293T cells were transfected with HA-LEF1 (**C**) or HA-Arc (**D**) and Flag-CHIP or Flag-CHIP domains. Then the protein interactions were detected by co-immunoprecipitation and immunoblotting as figures shown. All results were representative of three independent experiments.

Next, we sought to determine the detailed molecular mechanisms by which CHIP potentiates the activation of Wnt signaling. Firstly, we predicted the protein-protein interactions between CHIP and Arc or the downstream proteins of Wnt pathway by ZDOCK server. As shown in Table 1, CHIP might have strong interactions with Arc, β-Trcp, LEF1, or GSK3β. Co-immunoprecipitation assays (IP) revealed that CHIP specifically interacted with LEF1, Arc, and GSK3β, but had weak interactions with others (Fig. 3A). We have shown that CHIP promoted the activation of Wnt signaling by heightening the β-catenin nucleus accumulation, and had the strongest interaction with LEF1. It has been reported that LEF1 assists β-catenin to enter the nucleus and mediates the nuclear retention of β-catenin induced by Wnt3a^30^. So we focused on the interactions between CHIP and Arc or LEF1 instead of GSK3β. To identify the domains of CHIP responsible for the interaction with LEF1 or Arc, we expressed three CHIP deletion mutant proteins (Fig. 3B). CHIP could not interact with LEF1 or Arc after deleting the TPR domain (Fig. 3C, D), which suggested that the TPR domain of CHIP is essential for binding to LEF1 or Arc. Collectively, CHIP interacted with LEF1 or Arc, and its TPR domain was critical to the interactions.

**Table 1:** The protein-protein interactions between CHIP and Arc or the downstream proteins of Wnt pathway were detected by ZDOCK server.

Name	Accession number	Amino acids number	Template	Sequence identity (%)	Coverage range	ZDOCK score
Arc	AAF07185.1	396	6gse.1.A	97.48	206–364	1571.384
β-TrCP	NP_378663.1	605	1p22.1.A	100	175–581	1515.898
LEF1	AAH40559.1	386	2lef.1.C	98.68	270–345	1455.843
GSK3β	ABX89591.1	420	1j1b.1.B	100	23–386	1425.833
APC	AAA03586.1	2843	3nmz.1.A	100	326–736	1416.511
Rac1	CAB53579.5	192	1hh4.2.A	99.48	2–189	1409.991
Axin	AAI43245.1	778	1dk8.1.A	61.27	67–208	1338.895
CYLD	CAB93533.1	956	2vhf.1.A	100	583–956	1365.055
β-catenin	CAA61107.1	781	1v18.1.A	100	150–664	1361.005
Dvl-1	NP_001317240.1	695	1fsh.1.A	98.04	406–499	1216.274
CK1	CAA56710.1	374	6gzd.1.A	98.52	9–305	1176.247
TCF4	AAI25085	671	2ypa.1.B	81.01	561–633	1108.784
CHIP	AAH17178.1	303	2c2l.2.A	97.86	23–303	

The three-dimensional structure of proteins was obtained from an automated protein structure homology-modeling server of SWISS-MODEL. The higher ZDOCK scores were presented the stronger interactions between CHIP and proteins.

### CHIP increases the K63-linked ubiquitination of LEF1 and the K48-linked ubiquitination of Arc

**Fig. 4:**
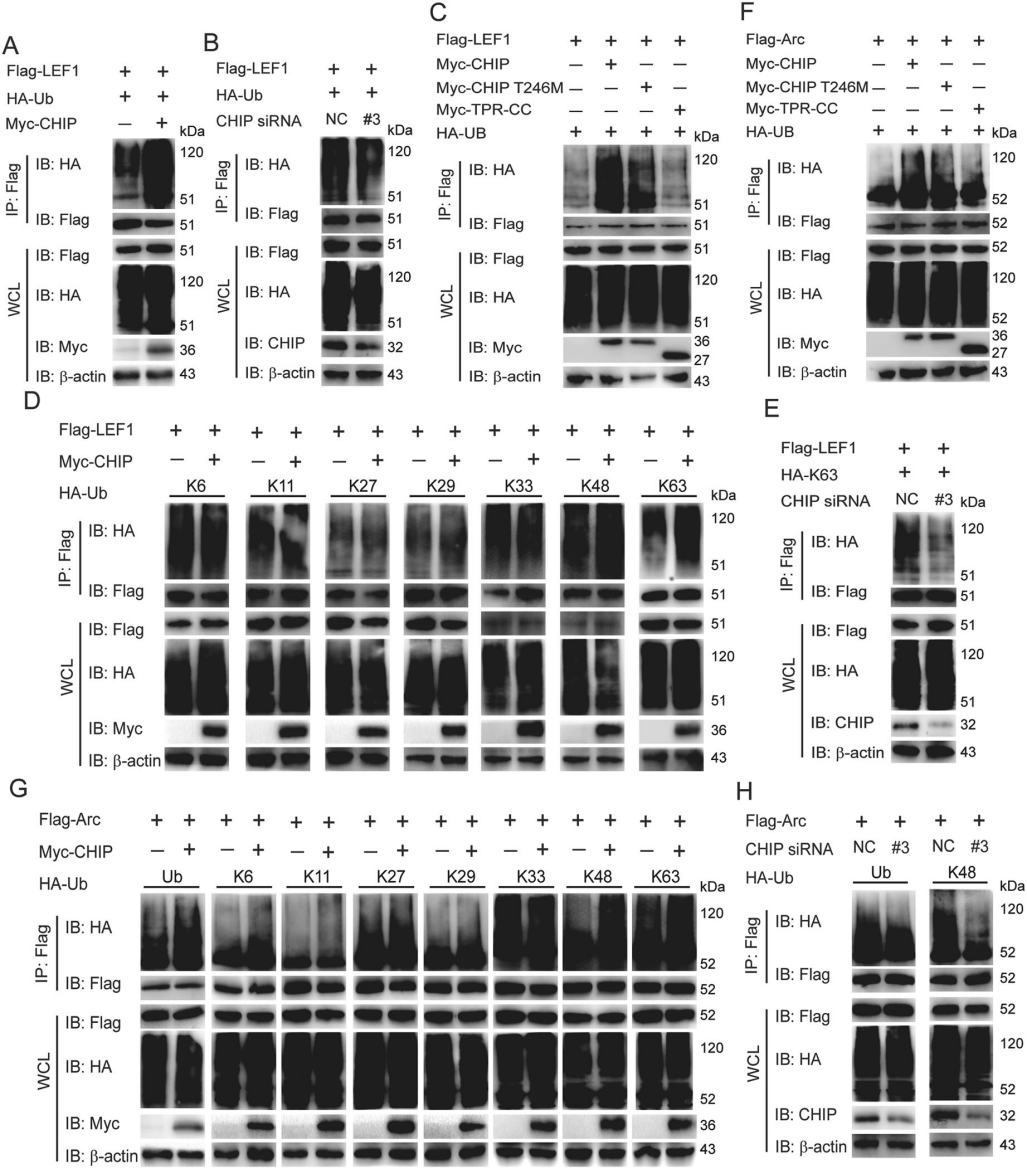
CHIP increases the K63-linked ubiquitination of LEF1 and the K48-linked ubiquitination of Arc. **A** CHIP promoted the ubiquitination of LEF1. Immunoprecipitation and immunoblot analysis were performed to detect the ubiquitination of LEF1 after co-transfection of HA-tagged ubiquitin, Flag-LEF1, and Myc-CHIP in HEK293T cells. **B** Knockdown of CHIP reduced the ubiquitination of LEF1. HEK293T cells were transfected with HA-tagged ubiquitin, Flag-LEF1, and CHIP-specific siRNA or control siRNA (NC) (40 nM), then the cell lysates were detected by co-immunoprecipitation and immunoblot. **C**–**E** HEK293T cells were transfected with indicated plasmids or CHIP-specific siRNA as figures shown. Whole cell extracts were immunoprecipitated with anti-Flag beads and blotted with anti-HA antibody. The inactive mutants of CHIP couldn’t enhance the ubiquitination of LEF1 as CHIP WT (**C**). CHIP increased the K63-linked ubiquitination of LEF1 (**D**). Knockdown of CHIP reduced the K63-linked ubiquitination of LEF1 (**E**). **F**–**H** Immunoprecipitation and immunoblot analysis were performed to detect the ubiquitination of Arc after co-transfection with indicated plasmids or CHIP-specific siRNA as figures shown. The inactive mutants of CHIP couldn’t enhance the ubiquitination of Arc (**F**). CHIP increased the K48-linked and K63-linked ubiquitination of Arc (**G**). Knockdown of CHIP reduced the ubiquitination, even the K48-linked ubiquitination of Arc (**H**). All results were representative of three independent experiments.

CHIP is an E3 ligase enzyme that consists of a TPR domain, a coiled-coil domain, and a U Box domain responsible for E3 ligase enzyme activity. So we next test whether CHIP affects the ubiquitination of LEF1. As showed in Fig. 4A, B, CHIP markedly enhanced the ubiquitination of LEF1, while knockdown of CHIP reduced the ubiquitination of LEF1. And we further found the enzymatically inactive mutant CHIP T246M or CHIP TPR-CC slightly or rarely enhanced the ubiquitination of LEF1 (Fig. 4C). We then examined which kinds of ubiquitination of LEF1 might be affected by CHIP. Overexpression of CHIP markedly enhanced the K63-linked ubiquitination of LEF1, but had little or no effect on the K6, K11, K27, K29, K33, or K48-linked ubiquitination (Fig. 4D). Consistent with this result, the knockdown of CHIP limited the K63-linked ubiquitination of LEF1 (Fig. 4E). These results indicated that CHIP potentiated the K63-linked ubiquitination of LEF1 through its E3 ligase activity.

Then we detect whether CHIP can affect the degradation of Arc via influencing Arc ubiquitination. The results showed that CHIP could enhance the ubiquitination of Arc, while the CHIP T246M and CHIP TPR-CC mutants had a reduced function (Fig. 4F). CHIP further markedly potentiated the K48-linked ubiquitination of Arc, and weakly enhanced the K63-linked ubiquitination, but not others (Fig. 4G). In addition, we confirmed this finding with CHIP-specific siRNA (Fig. 4H). These results indicated that CHIP promotes the degradation of Arc by heightening K48-linked ubiquitination of Arc, and also influenced the signal transduction by increasing the K63-linked ubiquitination of Arc.

### CHIP influences the interactions between β-catenin and LEF1 or GSK3β, as well as GSK3β and Arc

**Fig. 5:**
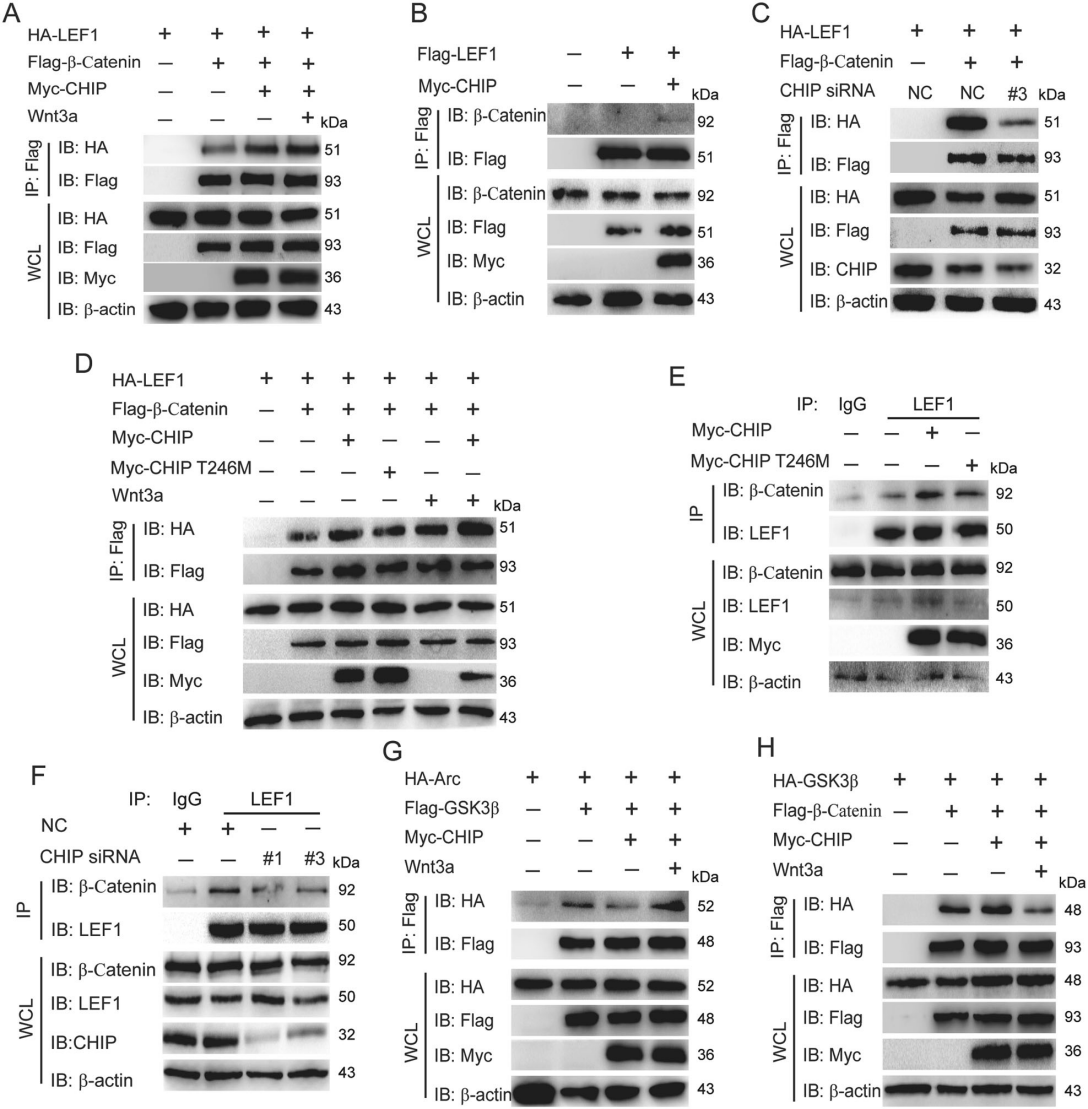
CHIP influences the interactions between β-catenin and LEF1 or GSK3β, as well as GSK3β and Arc. **A** CHIP enhanced the interaction between β-catenin and LEF1. Immunoprecipitation and immunoblot analysis of HEK293T cells which were transfected with HA-LEF1, Flag-β-catenin, and Myc-CHIP, and stimulated with Wnt3a (40 ng/ml) for 1 h. **B**–**D** HEK293T cells were transfected with indicated plasmids or CHIP-specific siRNA as figures shown. Whole cell extracts were immunoprecipitated with anti-Flag beads and blotted with anti-HA antibody. CHIP heightened the endogenous interaction between β-catenin and LEF1 (**B**). Knockdown of CHIP reduced the interaction between β-catenin and LEF1 (**C**). The inactive mutant of CHIP could not enhance the interaction between β-catenin and LEF1 (**D**). **E**–**F** Immunoprecipitation and immunoblot analysis were performed to detect the endogenous interaction between β-catenin and LEF1. The indicated plasmids or CHIP-specific siRNA were transfected as figures shown in SHSY5Y cells. Overexpression of CHIP enhanced (**E**), while knockdown of CHIP reduced (**F**) the endogenous interaction between β-catenin and LEF1. **G**–**H** HEK293T cells were transfected with various combinations of plasmids including HA-Arc, Flag-GSK3β (**G**), HA-GSK3β, Flag-β-catenin (**H**), and Myc-CHIP, or Myc-CHIP T246M, and stimulated with Wnt3a (40 ng/ml) for 1 h. Immunoprecipitation and immunoblot analysis of CHIP regulated the interaction between GSK3β and Arc or β-catenin. All results were representative of three independent experiments.

We have found that CHIP targeted and enhanced K63-linked ubiquitination of LFF1. Therefore, we want to know whether CHIP influences the interaction between LEF1 and β-catenin which then promotes Wnt signaling cascade. As shown in Fig. 5A, B, CHIP enhanced the interaction between β-catenin and LEF1, and the interaction was further heightened after Wnt3a treatment, while knockdown of CHIP decreased the interaction (Fig. 5C). Moreover, overexpression of CHIP T246M mutant couldn’t affect the interaction of β-catenin-LEF1 complex (Fig. 5D). To illuminate whether the heightened interaction of β-catenin and LEF1 was owing to the function of Wnt3a treatment in Fig. 5A, we detected the interaction between β-catenin and LEF1 as shown in Fig. 5D. Under Wnt3a stimulation, the interaction between β-catenin and LEF1 was enhanced, and the interaction was more strengthened with the presence of CHIP. We further found CHIP increased the endogenous interaction between β-catenin and LEF1, while CHIP T246M hardly influenced the interaction (Fig. 5E). Knockdown of CHIP reduced the endogenous interaction between β-catenin and LEF1 (Fig. 5F). These data suggested that CHIP enhanced the interaction between β-catenin and LEF1, and the interaction was further heightened with Wnt3a treatment.

It has been reported that Arc is phosphorylated, ubiquitinated, and subsequently degraded in GSK3α/β-dependent manner^28^. We hypothesized that CHIP participated in the degradation of Arc after GSK3β-mediated phosphorylation. Immunoprecipitation analysis revealed that CHIP reduced the interaction between Arc and GSK3β which might be convenient for the interaction of Arc and CHIP (Fig. 5G). Meanwhile, we found CHIP accelerated the interaction between GSK3β and β-catenin, which was associated with the activation of Wnt signaling (Fig. 5H). After the stimulation of Wnt3a, the interaction of Arc-GSK3β was enhanced, while the interaction of GSK3β and β-catenin decreased (Fig. 5G, H). It was in consistent with the enhanced interaction between β-catenin and LEF1 after Wnt3a treatment. Taken together, CHIP acts as a cargo to regulate Wnt signaling and the degradation of Arc by influencing the interactions between β-catenin and LEF1 or GSK3β, as well as GSK3β and Arc.

## Discussion

CHIP plays important role in cerebella homeostasis, cell proliferation, and tumor progression. It has been reported that the mutant of CHIP (p.T246M) lose E3 ligase activity, but maintains the features of the interactions with chaperones and chaperone-related functions which may lead to the occurrence of SCAR16^16,17^. But the detailed pathogenic mechanisms are still not clear.

Wnt signaling is an evolutionarily conserved pathway that controls multiple biological processes in embryonic and adult life. Growing evidences indicate that the dysregulation of Wnt signaling play a critical role in neurodegenerative diseases onset and development^22^. It has been reported that one of the pathogenesis of Alzheimer and Parkinson are the inactivation of Wnt signaling by regulating the activation of GSK3β or high leveled DKK1^31–34^. Although the role of CHIP in Wnt signaling may be related to the occurrence of SCAR16, the detailed regulatory mechanisms still needed to be studied. Our results showed that CHIP increases the protein level of β-catenin and active-β-catenin, enhances the expression of Wnt target genes, and further accelerates the nucleus accumulation of β-catenin detected by immunoblotting and fluorescence analysis, while knockdown of CHIP has the opposite effect. Compared to the CHIP WT, CHIP (p.T246M) mutant loses the ability to activate Wnt signaling. Moreover, knockout of CHIP inhibits the activation of Wnt signaling which further depresses cell proliferation and migration. We also confirm that Wnt signaling is inactivated in the rat model of SCAR16 induced by CHIP T246M mutant. These results indicate that CHIP may promote the activation of Wnt signaling which further influences the occurrence of SCAR16.

The regulatory mechanisms of β-catenin nuclear accumulation have not yet been elucidated. There are mainly two aspects influencing the nuclear accumulation of β-catenin: the transport and the retention in the nucleus by interacting with the nuclear pore complex^35,36^, and some nuclear localization signal-containing proteins, including FoxM1, IRS-1, and Rac1^37–39^, or Twa1/Gid8 or LEF1 which act as β-catenin nucleus retention factors in Wnt signaling^30,40–42^. Although they claim that LEF1 chaperones β-catenin into the nucleus is controversial, our data support that LEF1 facilitates β-catenin nuclear accumulation by increasing the nucleus retention. We demonstrate that CHIP can interact with LEF1, and markedly enhances the K63-linked ubiquitination of LEF1. The ubiquitinated LEF1 accelerates the interaction between β-catenin and LEF1, which is further heightened after Wnt3a treatment. Moreover, we verify that CHIP promotes the endogenous interaction between LEF1 and β-catenin, the CHIP (p.T246M) mutant does not affect the interaction, while knockdown of CHIP decreases the endogenous interaction of LEF1-β-catenin complex. Taken together, these results indicate that CHIP accelerates β-catenin nucleus accumulation by promoting interaction between LEF1 and β-catenin through heightening the K63-linked ubiquitination of LEF1.

Arc is dynamically regulated by neuronal activity serving as a master regulator of synaptic plasticity. It can regulate the endocytosis of synaptic AMPA receptors, which are involved in multiple forms of synaptic plasticity to influence learning-related behaviors by interacting with endosome-associated proteins like endophilin-2/3 and dynamin 2^23,43^. The alterations of Arc may have a positive or negative impact on the learning-related plasticity, which ultimately implicate neurological disease such as dementia, psychiatric disorders, and neurodegenerative disorders. The missense mutants in RNF216 (R660C and R694C) abolish the interaction, ubiquitination, and degradation of Arc, which contribute to SCAR16^25^. In this study, we also discovered CHIP affects the stability of Arc. Overexpression of CHIP reduces the protein level of Arc, while CHIP T246M hardly plays any role. CHIP can also interact with Arc, and enhances the K48 and K63-linked ubiquitination of Arc, while the enzymatically inactive mutant CHIP T246M and CHIP TPR-CC do not affect the ubiquitination of Arc. Since GSK3α and GSK3β is reported to influence synaptic plasticity and the plasticity of dendritic spines through catalyzing Arc phosphorylation and degradation^28^, we detect the interaction between Arc and GSK3β. CHIP reduces the interaction between Arc and GSK3β which may be convenient for the interaction between Arc and CHIP, then promotes the K48-linked ubiquitination and degradation of Arc. CHIP also accelerates the interaction between GSK3β and β-catenin which may restrict the nucleus accumulation of β-catenin. After stimulation of Wnt3a, the interactions between GSK3β and Arc or β-catenin are altered, which may promote the activation of Wnt signaling. Taken together, CHIP may act as a cargo to regulate the transduction of Wnt signaling and the stability of Arc to influence the occurrence of SCAR16.

In conclusion, we demonstrate that CHIP promotes the activation of Wnt signaling and regulates the degradation of Arc. Thus, our findings uncover the previously unknown mechanism of CHIP in signaling transduction, and may gain an insight into the molecular mechanisms by which the mutant of CHIP (p.T246M) results in the occurrence of SCAR16.

## Materials and methods

### Reagents, antibodies, and plasmids

Recombinant Human Wnt-3a (NP-149122) was purchased from an R&D system (Minneapolis, MN, USA).

Horseradish peroxidase (HRP)-anti-Flag (M2) (A8592), anti-β-actin (A1978), anti-rabbit-IgG-HRP (AP132P), and anti-mouse-IgG-HRP (AP308P) were purchased from Sigma (St. Louis, MO, USA). HRP-anti-hemagglutinin (12013819001) was purchased from Roche Applied Science (Switzerland, Basel). anti-c-Myc (HT101) and anti-β-tubulin (HC101) were purchased from TransGen Biotech (Beijing, China). anti-β-Catenin (8480) and anti-non-phospho β-Catenin (19807) were purchased from Cell Signaling Technology (Danvers, MA, USA). anti-LMNB1 (101237-T32) and anti-STUB1 (12496-R034) were purchased from Sino Biological (Beijing, China). anti-LEF1 (sc-374522) and anti-Arc (sc-17839) were purchased from Santa Cruz Biotechnology (San Diego, CA, USA).

Empty vector pcDNA3.1 was kindly provided by Dr Jun Cui (Sun Yat-sen University, Guangzhou, China). Target genes were cloned from A549 cDNA and subcloned into the pcDNA3.1 vector. The plasmids were confirmed by DNA sequencing at Sangon Biotech (Shanghai, China).

### Cell culture and transfection

Human embryonic kidney 293T (HEK293T) cells were kindly provided by Dr Jun Cui (Sun Yat-sen University). HT22 cells were kindly provided by Dr Fangxia Guan (Zhengzhou University). SHSY5Y cells were obtained from Cell Bank of the Chinese Academy of Sciences (Shanghai, China). L-Wnt3a cells were kindly provided by Dr Zongping Xia (The First Affiliated Hospital of Zhengzhou University, Zhengzhou, China). The brain tissues from rat models of SCAR16 (32 weeks) induced by the CHIP (p.T246M) mutant and WT rats (32 weeks) were kindly provided by Dr Yuming Xu (The First Affiliated Hospital of Zhengzhou University, Zhengzhou, China). These cells were cultured in DMEM (Hyclone, Logan, UT, USA) with 10% fetal bovine serum (BioIn, Israel), and 1% L-glutamine (Gibco, Carlsbad, CA, USA) at 37 °C in 5% CO_2_. Expression plasmids were transfected with Lipofectamine 2000 (Invitrogen, Carlsbad, CA, USA) according to the manufacturer’s instructions.

Wnt3a conditioned medium (Wnt3a CM) was obtained by spilling L-Wnt3a cells at the density of 1:10 and culturing in 10 ml DMEM medium without G418 for 4 days, and the medium was collected and filtered. Then the cells needed to add 10 ml fresh DMEM medium and cultured for another 3 days. The first batch and second batch of medium were collected, filtered, mixed, and stored at 4 °C. Ctrl-CM was obtained from L cells as the above method.

### Fluorescence analysis

HEK293T or HT22 cells were grown on coverslips, and then transfected with a green fluorescent protein (GFP)-tagged β-Catenin (β-Catenin-GFP) plus targeted plasmids or stimulated with ligands. The cells were stained with DAPI (Sigma) for 5 min. At last, the cells were fixed on the coverslips and observed under the fluorescence microscope (Olympus, Tokyo, Japan).

### MTT and migration assays

For MTT assay, the cells were seeded in 96-well plates at a density of 2 × 10^3^ cells per well for 24 h, and incubated with MTT (5 mg/ml, 20 μl) (Solarbio, Beijing, China) for 4 h, then added with 150 μl dimethyl sulfoxide (DMSO, Sigma). The absorbance of the samples was measured at 570 nm by Multiscan Spectrum (PerkinElmer EnSpire, USA).

For migration assay, the cells were seeded in 6-well plates at a density of 5 × 10^5^ cells per well. The monolayer cells were scratched using a 200 μl pipette tip, and then incubated and observed using a microscope (Olympus) after indicated times. The distances between the edges of scratch were measured to evaluate the capability of cell migration.

### Immunoblot analysis

Lysates from the transfected cells were extracted in 120 μl low-salt lysis buffer (50 mM Hepes pH 7.5, 150 mM NaCl, 1 mM EDTA, 1.5 mM MgCl2, 10% glycerol, 1% Triton X-100), supplemented with 5 mg/ml protease inhibitor (Thermo, Waltham, MA, USA) and phosphatase inhibitor Cocktail (Roche). The samples of 20 μl total proteins were subjected to SDS-PAGE and transferred onto PVDF membranes (Millipore, Schwalbach, Germany) with subsequent blocking using 5% skim milk (Solarbio). Membranes were incubated with specific antibodies, and detected using chemiluminescence (Millipore).

Cytosolic and nuclear fractions were obtained using Minute^TM^ Cytoplasmic and Nuclear Fractionation Kit for Cells (Invent Biotechnologies, Eden Prairie, MN, USA) according to the manufacturer’s instructions.

### Immunoprecipitation analysis

For immunoprecipitation experiments, whole-cell lysates were prepared as the method of immunoblot assay, followed by incubation with the anti-Flag agarose gels (Sigma) overnight at 4 °C. The beads were washed five times with low-salt lysis buffer, and then resuspended with 2 × SDS Loading Buffer (Solarbio), and boiled for 10 min. The released proteins were subjected to western blot analyses with the indicated antibodies.

For endogenous immunoprecipitation experiments, the extracted cell proteins were incubated with indicated antibodies overnight at 4 °C, and added with 20 μl Dynabeads protein G (Invitrogen) for another 2 h. Then the beads were washed and subjected to western analyses with the indicated antibodies.

### Real-time PCR

The total cellular RNA was isolated by TRIzol Reagent (Invitrogen) according to the manufacturer’s protocol. The first-strand cDNA was synthesized from total RNA using PrimeScript Frist Strand cDNA Synthesis kit (TAKARA, Otsu, Japan). Real-time PCR was performed with the SYBR Green qPCR Mix (TAKARA). *Myc*, *Cyclin D1*, and *Axin2* were analyzed by RT-PCR at 94 °C for 5 min, followed by 40 cycles at 94 °C for 20 s, at 56 °C for 20 s, at 72 °C for 20 s. The following specific primers are used for real-time PCR which is designed and synthesized by Sangon Biotech.

*hMyc* forward primer, 5′-CTTCTCTCCGTCCTCGGATTCT-3′

*hMyc* reverse primer, 5′-GAAGGTGATCCAGACTCTGACCTT-3′

*hCyclin D1* forward primer, 5′-AAGTGCGAGGAGGAGGTCTT-3′

*hCyclin D1* reverse primer, 5′-GGATGGAGTTGTCGGTGTAGA-3′

*hAxin2* forward primer, 5′-CCACCACTACATCCACCACC-3′

*hAxin2* reverse primer, 5′-TGCCTTTCCCATTGCGTTTG-3′

*hATCB* forward primer, 5′-GTCACCAACTGGGACGACAT-3′

*hATCB* reverse primer, 5′-TAGCAACGTACATGGCTGGG-3′

*mMyc* forward primer, 5′-CCCTATTTCATCTGCGACGAG-3′

*mMyc* reverse primer, 5′-GAGAAGGACGTAGCGACCG-3′

*mCyclin D1* forward primer, 5′-CCCTGGAGCCCTTGAAGAAG-3′

*mCyclin D1* reverse primer, 5′-TCATCCGCCTCTGGCATTTT-3′

*mAxin2* forward primer, 5′-AACCTATGCCCGTTTCCTCTA-3′

*mAxin2* reverse primer, 5′-GAGTGTAAAGACTTGGTCCACC-3′

*mATCB* forward primer, 5′-ATATCGCTGCGCTGGTCG-3′

*mATCB* reverse primer, 5′-TGGGGTACTTCAGGGTCAGG-3′

### Knockdown of CHIP by RNA interference

HEK293T or SHSY5Y were transfected with siRNAs using LipoRNAiMAX (Invitrogen) according to the manufacturer’s protocols. The sequences of STUB1 specific siRNAs are designed and synthesized by RiboBio as follows (Guangzhou, China).

#1 SenseSeq: CUGGAACAGUAUCGAGGAATT

AntiSeq: UUCCUCGAUACUGUUCCAGTT

#2 SenseSeq: CAACUUUGGGGAUGAUAUUTT

AntiSeq: AAUAUCAUCCCCAAAGUUGTT

#3 SenseSeq: GGAGAUGGAGAGUUAUGAUTT

AntiSeq: AUCAUAACUCUCCAUCUCCTT

### Statistical analysis

Data were compared between the different test groups using an unpaired, two-tailed Student’s *t*-test by the GraphPad Prism 5.0 software. All the experiments were repeated at least three times independently and the differences between groups were considered significant if *P < 0.05, **P < 0.01, and ***P < 0.001.

## Supplementary information

Figure S1
