# Supplementary figures and images for: CHIP promotes Wnt signaling and regulates Arc stability by recruiting and polyubiquitinating LEF1 or Arc
Source: Cell Death Discov. 2021 Jan 11;7:5. doi: 10.1038/s41420-020-00394-9 (PMC7801388; doi:10.1038/s41420-020-00394-9)

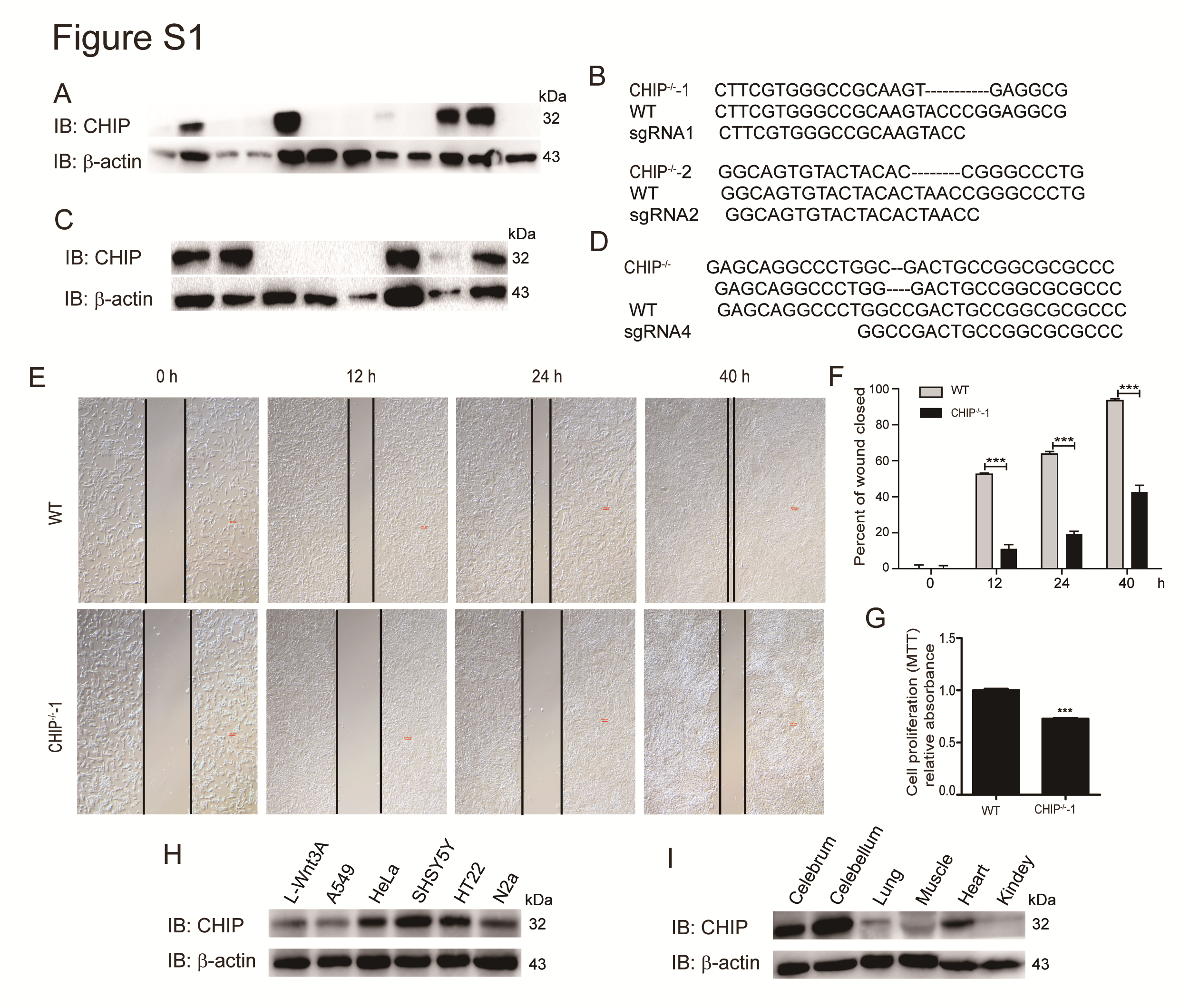

Supplement: Supplementary file 1 — Figure S1 [file 41420_2020_394_MOESM1_ESM.png]
